# Utilizing risk-controlling prediction calibration to reduce false alarm rates in epileptic seizure prediction

**DOI:** 10.3389/fnins.2023.1184990

**Published:** 2023-09-18

**Authors:** Galya Segal, Noam Keidar, Roy Maor Lotan, Yaniv Romano, Moshe Herskovitz, Yael Yaniv

**Affiliations:** ^1^Laboratory of Bioenergetic and Bioelectric Systems, Biomedical Engineering Faculty, Technion-Israel Institute of Technology (IIT), Haifa, Israel; ^2^Faculty of Medicine, Technion-Israel Institute of Technology (IIT), Haifa, Israel; ^3^Computer Science Department, Technion-Israel Institute of Technology (IIT), Haifa, Israel; ^4^Electrical and Computer Engineering Department, Technion-Israel Institute of Technology (IIT), Haifa, Israel; ^5^Department of Neurology, Rambam Health Care Campus, Haifa, Israel

**Keywords:** EEG, epilepsy, artificial intelligence, deep learning, seizure prediction, risk-controlling prediction, reliability

## Abstract

**Introduction:**

Epilepsy is a neurological disease characterized by sudden, unprovoked seizures. The unexpected nature of epileptic seizures is a major component of the disease burden. Predicting seizure onset and alarming patients may allow timely intervention, which would improve clinical outcomes and patient quality of life. Currently, algorithms aiming to predict seizures suffer from a high false alarm rate, rendering them unsuitable for clinical use.

**Methods:**

We adopted here a ***risk-controlling*** ***prediction*** calibration method called Learn then Test to reduce false alarm rates of seizure prediction. This method calibrates the output of a “black-box” model to meet a specified false alarm rate requirement. The method was initially validated on synthetic data and subsequently tested on publicly available ***electroencephalogram*** (EEG) records from 15 patients with epilepsy by calibrating the outputs of a deep learning model.

**Results and discussion:**

Validation showed that the calibration method rigorously controlled the false alarm rate at a user-desired level after our adaptation. Real data testing showed an average of 92% reduction in the false alarm rate, at the cost of missing four of nine seizures of six patients. Better-performing prediction models combined with the proposed method may facilitate the clinical use of real-time seizure prediction systems.

## 1. Introduction

Epilepsy is a prevalent condition characterized by abnormal brain activity and seizures (Fisher et al., 2005). The unpredictable nature of seizures is associated with severe injuries and reduced quality of life (Fisher et al., 2005). Early seizure prediction would allow patients to take precautions to increase their safety and even prevent the upcoming seizure by administration of abortive treatments, such as benzodiazepines (Appleton and Camfield, 2011).

The electroencephalogram (EEG) records of patients with epilepsy can be divided into three periods: an ictal state which is the seizure itself, an interictal state which is the time between seizures, and a preictal state defined as the time preceding each seizure and holds potential for seizure prediction within a specific time range (Rasheed et al., 2020). Seizure prediction involves triggering alarms during the preictal periods while avoiding false alarms during the interictal periods or the ictal state.

Several algorithms for seizure prediction have been developed over the past 50 years (Kuhlmann et al., 2018). Early research focused on identifying preictal patterns using linear features extracted from EEG signals. In 2013, the first clinical study was conducted by Cook et al. (2013) using an implanted warning system and demonstrated the feasibility of seizure prediction, although it was not yet clinically applicable. In the following years, numerous algorithms for seizure prediction were proposed, including machine-learning algorithms such as support vector machines, random forests, and k-nearest neighbors. Although many algorithms performed better than a random guess, they all had high false-positive (false alarm) rates.

In recent years, attempts to improve predictions were mainly focused on enhancing the prediction model. For example, Tsiouris et al. (2018) used a long short-term memory (LSTM) network for the first time to predict epileptic seizures, and Duy Truong et al. (2019) were the first to apply generative adversarial network (GAN) for seizure prediction. Although these methods succeeded in reducing false alarm rates, the false alarm rates remained excessively high for clinical utilization. Additionally, they lack the ability to adjust the false alarm rates according to specific clinical requirements. Furthermore, all these methods are model specific, which tightly couples the challenges of improving model performance and controlling the false alarm rate. In other words, reducing the false alarm rate requires improving the model itself.

The statistical literature is replete with methods that offer model-agnostic risk control for predictive models, ensuring rigorous statistical guarantees. These methods calibrate the outputs of an arbitrary model to limit a specific risk metric while potentially affecting other metrics. In the case of seizure prediction, these methods enable control over the false alarm rate of any seizure prediction model at a specified level, although it may come at the expense of sensitivity. Such methods include tolerance regions (Krishnamoorthy and Mathew, 2008), conformal prediction (Vovk et al., 1999), risk-controlling prediction sets (Bates et al., 2021), and the Learn Then Test method (LTT) (Angelopoulos et al., 2022), a recently published method that was shown to be superior to the others in both risk control and the variety of cases it can be applied to. The LTT method divides the ***risk-control*** problem into two well-known problems in statistics—computing ***p-values*** and ***multiple testing correction***, providing a simple method to control the expectancy of an arbitrary risk of any given black-box machine-learning model.

Here, we present our novel approach to false alarm rate reduction in seizure prediction. Our method is based on the LTT risk-controlling prediction calibration (Angelopoulos et al., 2022), with false alarm rate as the controlled risk. This method is independent of the prediction model used and allows improving the false alarm rate separately from improving the base prediction model. The LTT framework was adapted to a time series setting where the desired risk is defined over the time horizon. The adaptation was then validated on synthetic data and tested as a post-processing procedure on top of a deep-learning model predicting epileptic seizures on EEG recordings from patients with epilepsy. Implementation of the LTT framework applied to time series along with experiments on synthetic data is available at our GitHub repository: https://github.com/yyLabPhysAI/TS-LTT.

## 2. Methods

### 2.1. Data

#### 2.1.1. Synthetic

Many statistical methods are evaluated asymptotically when the amount of data go to infinity. When performance is guaranteed on real data where the number of samples is finite, the method is said to have ***finite sample guarantees***.

As a start, we validated that the adaption of the LTT method to a time series case yields the finite sample guarantees demonstrated in the original paper (Angelopoulos et al., 2022). This validation involved performing an analysis using synthetic data. Because the LTT calibration requires model outputs and corresponding ground truth labels as input, we simulated two binary time series. The first time series represented preictal intervals and was generated as a window of ones with zeros elsewhere. The second time series mimicked the behavior of a model by indicating the occurrence or absence of alarms over time.

To imitate the structure of the seizure prediction labels, we created signals with fixed-length windows of ones randomly spread and zeros elsewhere, representing the preictal periods of the seizures that occur occasionally. To construct a random series with this behavior, we first created a random series with mostly zeros and ones only occasionally, *Y*_*point*_[*t*], and then turned each of the sparse ones into a window of ones with a convolution operation to get the ground truth series *Y*[*t*]. Then, a “noisy oracle” was used to obtain synthetic model predictions, flipping the ground truth with a determined probability to achieve mostly good but not perfect predictions, *Y*_*pred*_[*t* ].

For a window length *w* and temporal probability of an event *p*, the synthetic data at any point are


(1) $$\begin{array}{l} Y_{point}\left[t\right]=𝕀\left[U\left(0,1\right)<p\right] \end{array}$$

where *U*(*a, b*) is a uniform distribution between *a* and *b*. Then, to transform each zero point into a window of ones, *Y*_*point*_[*t*] was convolved with a rectangular window, and an indicator function was applied again:


(2) $$\begin{array}{l} Y\left[t\right]=𝕀\left[Y_{point}*W\left(w\right)>0\right] \end{array}$$

where *W*(*w*) is a rectangular window of length *w*∈ℕ and * refers to the convolution operation. This step converts the sparse series created in the previous step (*Y*_*point*_, described in Equation 1) into a series of sparsely distributed windows of ones. Observe how the error is defined over the time horizon.

For *pw* < < 1, this construction yields a mostly zero time series with occasional windows of ones, resembling the structure of the seizure prediction label.

The synthetic model predictions are constructed as a *p*_*flip*_ oracle, being equal to the ground truth (*Y*[*t*], described in Equation 2) anywhere but certain points for which the label was flipped according to a determined probability *p*_*flip*_:


(3) $$\begin{array}{l} Y_{pred}\left[t\right]=\{\;\begin{array}{cc} Y\left[t\right] & U\left(0,1\right)<p_{flip}\\ not(Y\left[t\right]) & else \end{array} \end{array}$$

#### 2.1.2. Real data

We used non-invasive scalp EEG recordings from the Siena Scalp EEG Database (Goldberger et al., 2000; Detti et al., 2020),^1^ consisting of 34 long-term recordings from 15 patients. Each recording was at least 1-h long and included a minimum of 30 min of preictal intervals before each seizure. The dataset contained a total of 48 seizures.

**Figure 1:**
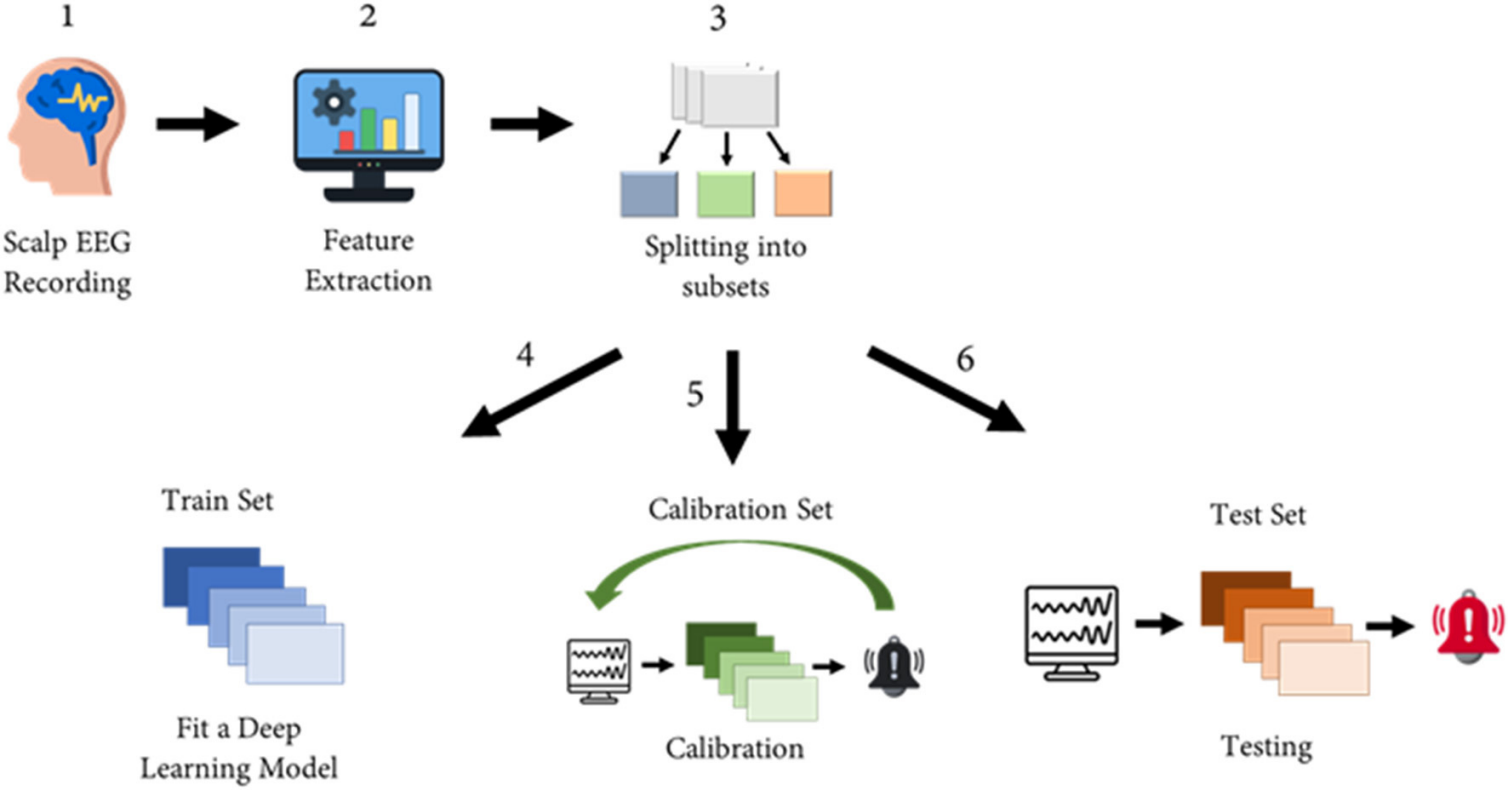
Schematic presentation of the data extraction pipeline. Step 1—Scalp electroencephalogram (EEG) is performed on patients with epilepsy. Step 2—EEG features are extracted from the recorded signals. Step 3—The data are divided into three subsets: training set, test set, and calibration set. Step 4—A deep learning model is trained on the training set to perform seizure prediction. Step 5—LTT calibration is performed based on the trained model using the calibration set. Step 6—The performance of the calibrated model is tested on the test set. This figure has been designed using icons from Flaticon.com and Vecteezy.com.

To enhance result resilience against overfitting, complete records were allocated to the train, calibration, and test sets. The test set followed a patient-specific approach, including patients with a minimum of three records, two of which had seizures. This biased selection ensured the representation of each patient for learning patient-specific patterns and calculating sensitivity (Figure 1). However, this split compromised the i.i.d assumption required for the LTT theorem.

### 2.2. Pre-processing

The raw EEG signal contained constant trend and high-frequency noise due to patient movement and acquisition noise. Therefore, frequencies below 0.5 Hz and above 75 Hz were filtered with a digital finite impulse response bandpass filter. Then, 10 features (see Table 1) were extracted from each channel in a rolling-window fashion to achieve an efficient representation of the signal. These features are widely used in neuroscience and were proven to be important in the field of seizure prediction and detection (Mormann et al., 2005; Kuhlmann et al., 2018; Boonyakitanont et al., 2020; Wong et al., 2023). Each feature was calculated over a 6-s window, with an overlapping window of 3 s.

**Table 1:** Features that were extracted from EEG signals.

**Feature**	**Short explanation**
Delta power	Fraction of spectral energy in the delta band in the interval of (0.4, 4) Hz (Mormann et al., 2005).
Theta power	Fraction of spectral energy in the theta band which is the interval of (4, 8) Hz (Mormann et al., 2005).
Alpha power	Fraction of spectral energy in the alpha band which is the interval of (8, 13) Hz (Mormann et al., 2005).
Beta power	Fraction of spectral energy in the beta band which is the interval of (13, 30) Hz (Mormann et al., 2005).
Gamma power	Fraction of spectral energy in the gamma band which is the interval of (30, 48) Hz (Mormann et al., 2005).
Spectral entropy	Quantifies the level of information or uncertainty to a possible outcome of a variable. We used spectral power to estimate entropy due to the fast computation (Boonyakitanont et al., 2020).
Detrended fluctuation analysis	Measures the statistical self-similarity of a signal and is useful for analyzing time series that appear to be long-memory processes, such as EEG signals (Boonyakitanont et al., 2020).
Hjorth mobility	Estimates the standard deviation of the power (Boonyakitanont et al., 2020).
Hjorth complexity	Estimates the mean frequency (Boonyakitanont et al., 2020)
Higuchi fractal dimension	Quantifies how a fractal pattern changes with a measured scale. It is associated with neurological conditions, such as epilepsy, Alzheimer's disease, and anxiety (Boonyakitanont et al., 2020).

The pipeline of filtering and feature extraction ran on a Ubuntu Linux machine with 80 CPU cores and 8 GPUs and took 48 h to complete with 80 worker multi-processing for the entire dataset.

To label the data, the preictal state was defined as the window of 60 min to 30 s before seizure onset, a period sufficient to warn the patient of an imminent seizure (Tsiouris et al., 2018). The window did not include the 30 s preceding a seizure, to prevent the model from performing seizure detection, rather than seizure prediction.

### 2.3. Models and training

The training setup was standard supervised learning. A convolutional learning neural network was chosen, as this method has a proven track record of success in solving a wide range of problems, specifically in tasks among other medical fields (LeCun et al., 2015). The input was fed to five convolutional layers, then flattened and passed on to fully connected layers. A binary cross-entropy loss (Keren et al., 2018), with Adam optimizer (Kingma and Ba, 2015) using cosine annealing learning rate scheduling (Loshchilov and Hutter, 2017), was chosen.

Training was performed on a GeForce RTX 2080 with 8 GB of RAM on a Ubuntu Linux server with 64 CPU cores. Model and training were implemented using PyTorch 1.10.1 (Paszke et al., 2019), Python 3.9 (van Rossum, 1995), and CUDA 11.3.^2^

The model was trained with a batch size of 512 samples, for 50 epochs using early stopping, with label noise (randomly replacing the current label with the opposite one) of probability 0.2. By introducing noise into the labels, the model was forced to consider a wider range of possible correct answers while training and was less likely to memorize specific patterns in the training data, which can prevent overfitting. The data preparation including feature extraction took ~ 48h and training the model about 2 min. Post-processing time and inference took <1 s over 24% of the data.

### 2.4. Risk-controlling prediction

Risk-controlling prediction (RCP) is a statistical method that allows a limitation of a specific risk measure of a model, e.g., the false alarm rate. It treats the predictive model as a black box, modifying only the model outputs, so it is not specific to a certain type of model. The predictions are transformed by a function *g*_λ_, which receives the model outputs as input and returns new predictions after calibration. It is dependent on a parameter λ, which depends on the probability of an alarm. For example, if we have a model that distinguishes between dog and cat images by assigning probabilities to each (e.g., the image is 70% likely to be a “dog” and 30% to be a “cat”) and we want to control the false prediction of the “dog” label, the post-processing can be a function that favors the “cat” label, e.g., choose “dog” only if the “dog” probability is higher than λ = 60% . If the threshold λ is calibrated using the proposed process, finite sample guarantees on the expectancy of the risk can be obtained. In our example, we can demand a false detection rate of “dog” to be < 5%. This is achieved by using calibration to get a λ value that will guarantee this condition when the results are averaged over many experiments.

Let $D_{N}={\left\{X_{i},Y_{i}\right\}}_{i=1}^{n}$ be a set of independent and identically distributed (i.i.d) examples in the dataset, s.t. $X_{i}\in X$, $Y_{i}\in Y$. Let A :X→Y be a model. For a parameter λ, let $g_{\lambda }\;:Y\;\to Y^{\prime }$ be a mapping function and let $T_{\lambda }≜A•g_{\lambda }$ (where ° is the standard function composition (*f*°*h*) (*x*) = *f*(*h*(*x*))) and let $R\left(\lambda \right)≜R\left(T_{\lambda }\right)$ be the risk function.

Let there be a subset $\hat{\Lambda }\subseteq \Lambda$ such that:


(4) $$\begin{array}{l} \forall \hat{\text{λ}}\in \hat{\Lambda }:\mathbb{P}\left[R\left(\hat{\text{λ}}\right)\leq \alpha \right]\geq 1-\delta . \end{array}$$

If the equation above is satisfied, we can say that $\forall \hat{\lambda }\in \hat{\Lambda }$, $T_{\lambda }$ is an (α, δ)***-*** RCP. Namely, for the λ values that satisfy this condition, the risk (e.g., false alarm rate) is lower than a desired value α (e.g., less than one false alarm per hour) with a probability of at least 1−δ, where the latter probability is defined over the calibration set. Between the “legal” values of λ, we can choose a value that provides the best performance in a different metric, optimizing the tradeoff between two required properties of the system.

In the first presentation of the LTT method (Angelopoulos et al., 2022), the proof was provided for a claim that given valid *p*-values for the ***null hypotheses*** that each λ value does not control the risk at level α, applying a ***familywise error rate*** (FWER) controlling ***multiple hypothesis correction*** (MHC) with an FWER of δ yields an (α, δ)-RCP.

### 2.5. Time-series adaptation

To adapt the LTT framework to time-series data, a *g*_λ_ function that relates to the sequential nature of the input was found. The function includes two steps of temporal aggregation. First, a window majority vote (i.e., setting the result of the entire window to be positive only if it has enough positives within) is performed with windows of length *w* so only windows with more than λ fraction of positives are considered positive:


(5) $$\begin{array}{l} Y_{MV}\left[t\right]\\ =𝕀\left[\frac{Y\left[t-w+1\right]+Y\left[t-w+2\right]+\ldots +Y\left[t\right]}{w}>\lambda \right].\; \end{array}$$

Then, the time series is ***max-pooled*** over the lower temporal resolution, with the *k* pooling rate defined as


(6) $$\begin{array}{l} Y_{pooling}\left[n\right]\\ =\max\left(Y_{MV}\left[\left(n-1\right)\cdot k\right],Y_{MV}\left[\left(n-1\right)\cdot k+1\right],\ldots ,Y_{MV}\;\left[n\cdot k\right]\right) \end{array}$$

with the majority vote (Equation 5) intended to add robustness against noise in the model predictions and the pooling (Equation 6) to mitigate the effect of point mistakes.

When fixing *w, k* and calibrating only λ, $T_{\lambda }$ satisfies the LTT theorem conditions for i.i.d samples.

The central limit theorem (CLT) was used to create *p*-values from the metrics calculated in the calibration set. For MHC, two methods were compared; first, we used the Bonferroni correction as it works under arbitrary dependence between the hypotheses. Then, as the correction seemed to be too strict (e.g., only very small *p*-values were considered significant), yielding over-tight control of the risk, a less restrictive method was implemented. Considering the fact that in our $T_{\lambda }$, higher values of λ always result in stricter control of the false alarm rate, our use case satisfies the *p*-value monotonicity requirement of the ***fixed-sequence testing*** (FST) method (Angelopoulos and Bates, 2021). Under this requirement, the FST provides the same guarantee on the FWER with less restrictive conditions on the *p*-value significance. While many more statistically powerful MHC methods than the Bonferroni correction exist, some do not utilize the monotonicity of *p*-values (Holm, 1979), others control only the false discovery rate and not the FWER and therefore do not satisfy the LTT theorem conditions (Benjamini and Hochberg, 1995), and some, e.g., sequential graphical testing (Angelopoulos et al., 2022), are sequential FWER controlling and even less restrictive than FST, but with the price of being more complicated.

### 2.6. Performance statistics

The performance of the deep learning model was evaluated by two measures: sensitivity and false alarm rate. Tailored definitions of both metrics were used to allow the metrics to reliably reflect the clinical use of such a predictive system.

#### 2.6.1. Sensitivity

Sensitivity was intuitively defined as: “Out of all seizures that occurred, how many seizures were correctly predicted?” A single alarm raised during the preictal period is sufficient to warn the patient of an upcoming seizure; the number of alarms raised during the preictal state does not matter clinically, so long as at least one alarm was raised. Therefore, a ***true positive*** (TP) was defined as seizures for which at least one alarm was raised during the preictal state. ***False negatives*** (FN) were defined as seizures for which no alarms were raised. The sensitivity was calculated as the number of true positives divided by the total number of seizures that occurred:


(7) $$\begin{array}{l} Sensitivity=\frac{TP}{TP+FN} \end{array}$$

#### 2.6.2. False alarm rate

An alarm raised at any time other than the preictal state was considered a false positive. The false alarm rate was calculated as the number of false positives divided by the number of non-preictal hours in the record:


(8) $$\begin{array}{l} False\;Alarm\;Rate=\frac{FP}{non\;preictal\;hours} \end{array}$$

This is a conservative definition that will consider both “nearly preictal” (e.g., 61 min before the event) and ictal periods as false alarms. We chose this approach for two reasons. First, the model was trained to perform prediction within a strict time interval. Second, due to the short record durations, in some cases, less conservative approaches will not have long enough non-preictal times to stably calculate the metric.

## 3. Results

### 3.1. Synthetic data

The post-processing function and the LTT implementation were validated on the synthetic data described above for different risk control limits (α = 0.001, 0.005, 0.01, 0.05, 0.1, 1), with and without the pooling step and with the two methods for multiple hypothesis correction. Synthetic data were generated as described in the methods section, with 10,000 sequences of length 1,000 for each experiment, and with prediction window labels of length 8. In all experiments, the post-processing window length was 10 and, when applicable, the pooling length of 10. Aggregated results of 200 experiments for each α are presented in Table 2 for the pooling and non-pooling comparison and in Table 3 for the Bonferroni correction vs. ***fixed-sequence testing*** MHC methods.

**Table 2:** Comparison of synthetic data results with and without pooling at different α levels.

	**False alarm rate (**hr**** ^**−1**^ **)** ^*^		**Accuracy ( )** ^*^		**λ**	
**α**	**Without pooling**	**With pooling**	**Without pooling**	**With pooling**	**Without pooling**	**With pooling**
0.001	6.7e-05 ± 4.1e-06	3.4e-06 ± 2.6e-06	0.93 ± 0.00032	0.87 ± 0.00064	0.81	0.81
0.005	0.0012 ± 2e-05	0.00015 ± 0.00023	0.97 ± 0.00017	0.92 ± 0.014	0.61	0.69
0.01	0.0012 ± 2.2e-05	0.00073 ± 3.6e-05	0.97 ± 0.00016	0.96 ± 0.00032	0.61	0.61
0.05	0.0053 ± 5.3e-05	0.0048 ± 0.00011	0.98 ± 0.00012	0.98 ± 0.00021	0.51	0.51
0.1	0.0053 ± 4.6e-05	0.0048 ± 0.0001	0.98 ± 0.00011	0.98 ± 0.00021	0.51	0.51
1	0.0053 ± 4.7e-05	0.0048 ± 9.8e-05	0.98 ± 0.00011	0.98 ± 0.00018	0.51	0.51

^*^Results are presented as mean ± standard deviation.

**Table 3:** Comparison of synthetic data results with different multiple hypothesis correction methods and α levels.

	**False alarm rate (**hr**** ^**−1**^ **)** ^*^		**Accuracy ( )** ^*^		***λ***	
**α**	**Bonferroni**	**FST**	**Bonferroni**	**FST**	**Bonferroni**	**FST**
0.001	3.2e-06 ± 2.6e-06	3.4e-06 ± 2.6e-06	0.87 ± 0.0027	0.87 ± 0.00064	0.81	0.81
0.005	6.5e-05 ± 1.2e-05	0.00015 ± 0.00023	0.92 ± 0.00046	0.92 ± 0.014	0.7	0.69
0.01	0.00063 ± 0.00023	0.00073 ± 3.6e-05	0.95 ± 0.015	0.96 ± 0.00032	0.62	0.61
0.05	0.0048 ± 0.0001	0.0048 ± 0.00011	0.98 ± 0.00019	0.98 ± 0.00021	0.51	0.51
0.1	0.0048 ± 9e-05	0.0048 ± 0.0001	0.98 ± 0.00021	0.98 ± 0.00021	0.51	0.51
1	0.0048 ± 0.0001	0.0048 ± 9.8e-05	0.98 ± 0.00019	0.98 ± 0.00018	0.51	0.51

^*^Results are presented as mean ± standard deviation.

A comparison of the pooling and the non-pooling post-processing model results found that pooling yields a better false alarm rate (*p* < 0.001 for all α values tested), sometimes with decreased sensitivity. The MHC method comparison showed that FST yielded a false alarm rate that was closer to the determined risk limit (α), but that was still controlled. Note that for some values of the risk limit, both methods behaved exactly the same, as the change in significance threshold did not change the allowed λ values.

### 3.2. Real data

**Figure 2:**
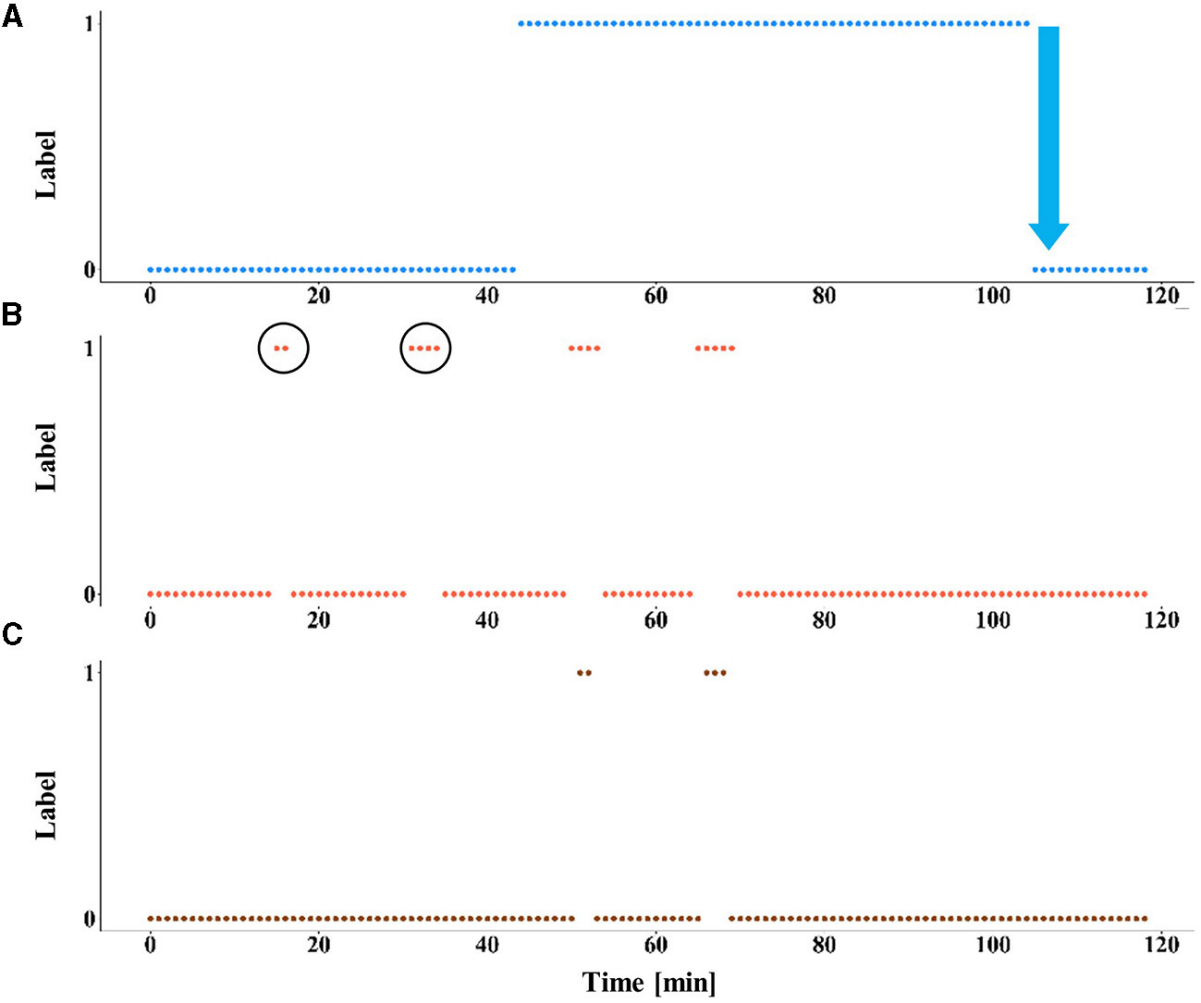
The effect of LTT calibration on model predictions. An example of the results of the prediction model, with and without LTT on patient 10 (record number 2). **(A)** The preictal state, defined as the interval from 60 min to 30 s before seizure onset is labeled as 1. Seizure onset is marked by a blue arrow. **(B)** The predictions of the deep learning model without LTT. False alarms are circled. **(C)** The predictions with LTT post-processing.

The model was tested with and without LTT post-processing on the test set. Pooling was applied, such that a prediction was given every 60 s. The test set included seven records from six different patients (Table 4). The false alarm rate was lower in all records in the test set, with a weighted average of 93.5% reduction in false alarm rate compared with the raw predictions, with the cost of missing four of nine seizures. A reduction of 100% in the false alarm rate (no false alarms) was observed in four of six patients (see Figure 2 for an example). Among these four patients, all seizures of two patients were still correctly predicted. One patient had two seizures, of which one was now predicted and the other was missed. The remaining patient had only a single seizure in the recording, which was missed.

**Table 4:** Statistic matrix on EEG data.

**Record metadata**						**Sensitivity (%)**		**False alarm rate (** ***h*** ^**−1**^ **)**		**False alarm rate reduction (%)**
**Test batch index**	**Number of items**	**Record duration (H:m:s)**	**PID**	**RID**	**Number of seizures**	**Raw**	**LTT**	**Raw**	**LTT**	
1	2,217	1:50:51	8	2	1	100	0	2.7	0	100
2	2,388	1:59:24	10	2	1	100	100	4.8	0	100
3	2,604	4:22:27	11	3	1	100	0	4.2	0	100
4	5,249	4:22:27	11	4	1	100	100	6.6	0	100
5	2,957	2:27:51	12	2	3	100	33	5.8	2.1	64
6	2,803	2:20:09	13	2	1	100	100	5.7	0	100
7	2,754	2:17:42	14	2	1	100	100	9.1	1.6	83
Total Test	20,972	19:40:51	–	–	9	100	55	5.60	0.45	93.51

Results of the prediction model, with and without LTT, are shown on the test set. The test set included seven records from six different patients. The model performance was evaluated by the sensitivity and the false alarm rate. Those were calculated for each record in the test set, with and without the LTT post-processing calibration. The percentage of reduction in false alarm rate is also shown for each record. The time duration of each record, along with the number of decisions made by the model during each record, is also shown.

## 4. Discussion

This study used the LTT framework to reduce false alarms in real-time series data. The time-series adaptation of the LTT framework maintained the finite sample guarantees in synthetic data experiments. Real data experiments applying the time-series LTT to an epileptic seizure prediction deep learning model showed a significant reduction in false alarm rate at a cost of sensitivity reduction.

The addition of LTT dramatically reduced the false alarm rate in all patients in the test set, but some with a cost of a large reduction in sensitivity. Manual comparison of the calibrated against the original model predictions found that records suffering major sensitivity reduction had alarms distributed evenly between interictal and preictal windows. Thus, we attribute the original high sensitivity to the model's low specificity, i.e., in these records, the sensitivity loss in calibration merely uncovered poor model performance and did not reflect a compromise. Longer recordings from those patients may improve the algorithm's performance.

While the calibration significantly improved model performance on some records, the results were still far from clinically relevant. For example, the calibrated model gave an average of 2.1 false alarms per hour in patient 12 and failed to predict 4 out of 9 seizures. Future study should focus on achieving a model with better performance. As deep learning models are known to be “data-hungry”, a larger dataset is the key to significant performance gain in this task. Nevertheless, our study showed that LTT can be very useful in improving the false alarm rate of a prediction model in a time series. A larger cohort would facilitate a more detailed investigation of the LTT adaptation, allowing for optimization of the MHC and *p*-value estimation methods selection, and the detection of subtle effects on the results.

As stated by Angelopoulos et al. (2022), not all risks can be controlled at any requested level. During the calibration process, this problem was encountered when trying to control the false alarm rate. When attempting to reduce the risk, we got an empty set of controlling λ values. This can be explained by the model performance, demanding low false alarm rates from an inaccurate model results in a calibration process for which no λ is high enough, as even with the highest values, false alarms still occur. Also, because the dataset was small and had to be divided into three subsets, the calibration set included only five records. Estimating *p*-values based on the CLT using a small sample size, resulted in an inaccurate calibration (the CLT provides only asymptotic convergence and no finite sample guarantees), and the false alarm rate could only be controlled over large values. Increasing the amount of data will not only improve the model performance but will also improve the calibration process and allow for better control over the false alarm rate.

The use of the fixed-sequence testing, a less conservative MHC method than the Bonferroni correction, allowed for tighter control of the risk; the risk values were controlled but not over-controlled; i.e., actual values of the risk were closer to the limit required, without compromising other metrics.

The LTT theorem requires calibration samples to be independent and identically distributed (i.i.d) to provide risk control. In the synthetic data experiment, the samples were independently generated by a pseudorandom number generator and therefore satisfied the i.i.d requirements. The results, as expected, showed perfect risk control over the false alarm rate. Although this construction ensures that the LTT theorem requirements are met and enables empirical validation of the adaptation and implementation, the simplistic nature of the surrogate data does not fully capture the complexity of real model predictions. Consequently, some phenomena may arise in real data experiments that were not observed in this synthetic data experiment.

However, in the real data experiment, random sampling was not possible due to the small dataset size. Interestingly, risk control was still achieved for all patients. This result may be attributed to the conservative nature of the *p*-value estimation and MHC methods used. With larger datasets, samples can be randomly assigned into groups, forming an i.i.d. calibration set and formally satisfying the LTT theorem.

Although a patient-independent approach would have been preferable for demonstrating a clinically usable seizure prediction system, only a patient-specific approach provided predictions of sufficient quality to assess the effect of the LTT. As the LTT treats the model as a black box, the conclusions regarding risk control remain applicable to models trained using a patient-independent approach. Future research could utilize the LTT calibration with an improved model to develop a robust and clinically useful prediction system with well-defined risk limits.

The LTT was adapted to a time series using two aggregation functions for the model predictions over time: majority vote and pooled majority vote. Both methods yielded similar results, with the pooled version providing lower false alarm rates (stricter control of the risk), but also slightly lower sensitivity and accuracy. As the pooled version creates a more stable prediction (model prediction needs to be more decisive to change the output), it might be advantageous for some applications, even if performing significantly worse for the lower α values than the non-pooled version.

Summarizing the limitations of the study, a larger cohort would have facilitated additional enhancements to the LTT adaptation and potentially resulted in a more effective prediction model. Such a model could have enabled a patient-independent train-test approach, thus preserving the i.i.d assumption of the LTT theorem. These limitations do not undermine the conclusion regarding risk control but suggest that the method can still be further improved.

Future studies on seizure prediction can potentially benefit from the proposed method to improve robustness and meet clinical requirements. Additionally, further research using larger datasets could optimize the method and enhance control over the false alarm rate.

## 5. Conclusion

Learn Then Test calibration, with a majority vote and pooling aggregation post-processing function, successfully controlled the false alarm rate of epileptic seizure prediction while compromising sensitivity. Better-performing base models may improve risk control.

## Data availability statement

The Siena EEG dataset is available at: https://physionet.org/content/siena-scalp-eeg/1.0.0/.

## Ethics statement

The studies involving humans were approved by Neurology Department of Siena University Hospital, Italy. The studies were conducted in accordance with the local legislation and institutional requirements. The participants provided their written informed consent to participate in this study.

## Author contributions

GS, NK, and RL conceived the research, developed the algorithm, and drafted the manuscript. GS and NK performed the experiments. YY designed and supervised the research. MH provided clinical guidance for the research. YR provided mathematical guidance for the research. YY, MH, and YR edited and revised the manuscript. All authors approved the final version.

## Footnotes

^1^Siena Scalp EEG Database v1.0.0.

^2^CUDA Toolkit 11.3 Downloads | NVIDIA Developer Available online at: https://developer.nvidia.com/cuda-11.3.0-download-archive (accessed October 3, 2022).
